# Assessing the Capability and Potential of LiDAR for Weed Detection

**DOI:** 10.3390/s21072328

**Published:** 2021-03-26

**Authors:** Nooshin Shahbazi, Michael B. Ashworth, J. Nikolaus Callow, Ajmal Mian, Hugh J. Beckie, Stuart Speidel, Elliot Nicholls, Ken C. Flower

**Affiliations:** 1 UWA School of Agriculture and Environment, The University of Western Australia, Crawley, Stirling Highway, WA 6009, Australia; nooshin.shahbazi@research.uwa.edu.au (N.S.); mike.ashworth@uwa.edu.au (M.B.A.); nik.callow@uwa.edu.au (J.N.C.); hugh.beckie@uwa.edu.au (H.J.B.); 2 Australian Herbicide Resistance Initiative, The University of Western Australia, Crawley, Stirling Highway, WA 6009, Australia; 3 UWA School of Computer Science and Software Engineering, The University of Western Australia, Crawley, Stirling Highway, WA 6009, Australia; ajmal.mian@uwa.edu.au; 4 Stealth Technologies, 138 Churchill Avenue, Subiaco, WA 6008, Australia; stuart@stealthtechnologies.com.au (S.S.); elliot@stealthtechnologies.com.au (E.N.); 5 UWA Institute of Agriculture, The University of Western Australia, Crawley, Stirling Highway, WA 6009, Australia

**Keywords:** light detection and ranging (LiDAR) sensors, weed detection, target size, scanning distance, target orientation

## Abstract

Conventional methods of uniformly spraying fields to combat weeds, requires large herbicide inputs at significant cost with impacts on the environment. More focused weed control methods such as site-specific weed management (SSWM) have become popular but require methods to identify weed locations. Advances in technology allows the potential for automated methods such as drone, but also ground-based sensors for detecting and mapping weeds. In this study, the capability of Light Detection and Ranging (LiDAR) sensors were assessed to detect and locate weeds. For this purpose, two trials were performed using artificial targets (representing weeds) at different heights and diameter to understand the detection limits of a LiDAR. The results showed the detectability of the target at different scanning distances from the LiDAR was directly influenced by the size of the target and its orientation toward the LiDAR. A third trial was performed in a wheat plot where the LiDAR was used to scan different weed species at various heights above the crop canopy, to verify the capacity of the stationary LiDAR to detect weeds in a field situation. The results showed that 100% of weeds in the wheat plot were detected by the LiDAR, based on their height differences with the crop canopy.

## 1. Introduction

Weeds pose a significant annual threat to global dryland cropping. In order to combat this threat, growers have developed numerous herbicidal and non-herbicidal weed control techniques. Weed control poses one of the greatest costs to growers as the majority of applications is by herbicide applied uniformly across entire fields [1]. One solution in reducing grower input costs, whilst maintaining effective control of weed-infested areas is the use of site-specific weed management (SSWM), a form of precision agriculture (PA) [2]. SSWM has lower environmental impacts with less herbicide being used at a landscape scale [3]. In order to spatially apply weed control treatments efficiently, growers must have access to technologies that can ascertain the spatial distribution of target weed species within the field [4]. Since the manual recording of crop field data is labor-intensive, there is a need for automation to achieve these SSWM outcomes. New automated technologies, using remote or proximal sensors have been promoted [5]. They include airborne remote sensing which has been demonstrated to detect high-density weed patches [6,7], drone-mounted sensors that have been used for multiple applications at the individual plant-scale [8,9] and commercialized tractor and spray boom-mounted multispectral sensor technology [10]. However, current techniques require considerable time for data processing [11] and are focused on an actively growing crop and they target within-season weed growth with herbicides. Furthermore, weather conditions that cause multiple scatterings such as fog, rain, dust [12] or cloudy conditions [13] can affect the quality of the results from infrared cameras.

With crop management operations occurring routinely within a field, there is scope for the ongoing collection of data using ground-based detectors mounted to field equipment. Ground-based mapping systems have shown good potential for weed mapping in wide-row crops [11,14,15], with vision technology capable of discriminating weed species based on their texture, shape and color [16]. However, the accuracy of these technologies is significantly reduced when weeds and crops grow larger, and their leaves start to overlap [17]. Limitations of ground-based sensors such as high cost and their ability to distinguish between weeds and crops only in inter-row areas have led to testing more economical sensors for SSWM systems. For example, Zaman et al. [18] and Andújar et al. [19] developed a new weed detection system by using ultrasonic sensors. These systems are low-cost, but they have limitations such as a small field of view and several sensors are required on the machinery to cover the field area [5]. Existing systems are focused on detection and mapping the actively growing weeds within the crop and the application of within-season herbicide treatment. There exists an as yet unexploited opportunity, to leverage technology when passing over the senesced crop at harvest to map above-crop weeds and develop SSWM strategies around herbicide or non-herbicide methods to target the weed seed bank based on mapping the late-season weeds.

Light detection and ranging (LiDAR) sensors are a powerful and relatively low-cost mapping technique [20] with strong scanning capabilities and a wide field of view [5,21,22]. Development of LiDAR technology opens new horizons for detecting taller plants in a canopy at late phenological stages [23]. Andújar et al. [17] used a terrestrial LiDAR-based system to detect the weed species in the inter-row area of maize (*Zea mays* L.) crops at early phenological stages. Andújar et al. [5] continued that study by adding two indices of height and reflection values to discriminate maize crops and weeds from the soil surface in the row and inter-row area at the early stages of weed and crop growth. Friedli et al. [24] and Jimenez-Berni et al. [25] assessed LiDAR performance for estimating wheat (*Triticum aestivum* L.) canopy height. These studies showed a strong correlation between the actual height of the canopy and LiDAR-estimated heights. Guo et al. [26] studied LiDAR performance for measuring wheat plant heights during the entire wheat growth stages. This study showed that the wheat plants could be detected as low as 0.18 m in height when the LiDAR scanners were located at the four corners of the study area. Moreno et al. [27] reported high accuracy of the LiDAR sensors for detecting plant features such as height and branch volume of vineyard *(viticulture)* crops at the early stages of plant growth, which showed the capability of these sensors at detecting hard to detect targets such as defoliated crops. However, the effectiveness of the LiDAR systems for detecting and mapping weeds that grow taller than the crop canopy and persist in the crop until harvest is still unknown. Using manual height measurements, Shahbazi et al. [28] reported that a number of common weeds grow taller than crops, so could potentially be differentiated based on their height. Weeds mapped at late phenological stages indicate where the weeds were not controlled, due to ineffectiveness of the herbicide, herbicide resistance or not being sprayed [29]. Some of the persistent weed species that grow taller than the surrounding crops include cannabis (*Cannabis sativa*) and brown mustard (*Brassica juncea* (L.) *Czern*) weeds in spring wheat in Western Canada [30], annual sow thistle (*Sonchus oleraceus* L.) in wheat crops in Eastern Australia [31] and wild oat (*Avena fatua* L.), wild radish (*Raphanus raphanistrum* L.) and sow thistle in wheat, barley (*Hordeum vulgare* L.), lupin (*Lupinus angustifolius* L.) and chickpea (*Cicer arietinum* L.) crops in the central wheat-belt region of Western Australia [28].

It is considered plausible that LiDAR sensors can detect weeds in crops by differentiating plant height. The objective of this study was to determine the width, height, and distance limits that a 3D scanning LiDAR (SICK AG, Melbourne, Australia) could detect the ‘weed’ target. Two trials were conducted using wooden round rod targets of known dimensions, with the aim of determining the detection limits (target diameter and differential height of the LiDAR when positioned at a range of vertical angles and scanning distances). A third trial was performed in a wheat plot where the LiDAR was used to scan different target weed species at various heights taller than the crop canopy, to verify the capacity of the stationary LiDAR to detect weeds in a field situation.

## 2. Materials and Methods

### 2.1. Setup of Trials

#### 2.1.1. Scanning Lines of Rods

An MRS6000 LiDAR (SICK AG, Melbourne, Australia) system was used for this study. It had 24 scanning layers of vertical pixels across the scans, for gap-free and reliable detection, and a scanning frequency of 10 Hz. The LiDAR had an angular resolution of 0.13°, with a maximum horizontal and vertical aperture of 120° and 15°, respectively. The working range of the LiDAR was from 0.50 to 200 m with the ambient operating temperature of −20 °C to +60 °C. The source of light was an infrared laser of 905 nm, with distances calculated based on the return times of the beams. The light spot divergence for the MRS6000 can be calculated by “light spot divergence [rad] × Distance [mm] + light spot width at the n device cover [mm] = light spot width [mm]” [32]. The light spot size at the front screen of the device and at a distance of 25 m was 0.25 mm (horizontal) × 8.0 mm (vertical) and 0.05 m (horizontal) × 1.64 m (vertical), respectively [32]. The LiDAR was connected to a Piksi Multi Real-Time Kinematic (RTK) global positioning system (GPS) receiver and base station, with centimeter accuracy. The LiDAR calibration prior to the scans showed +2° vertical divergence of the beams, which was taken into account in the data processing.

**Figure 1:**
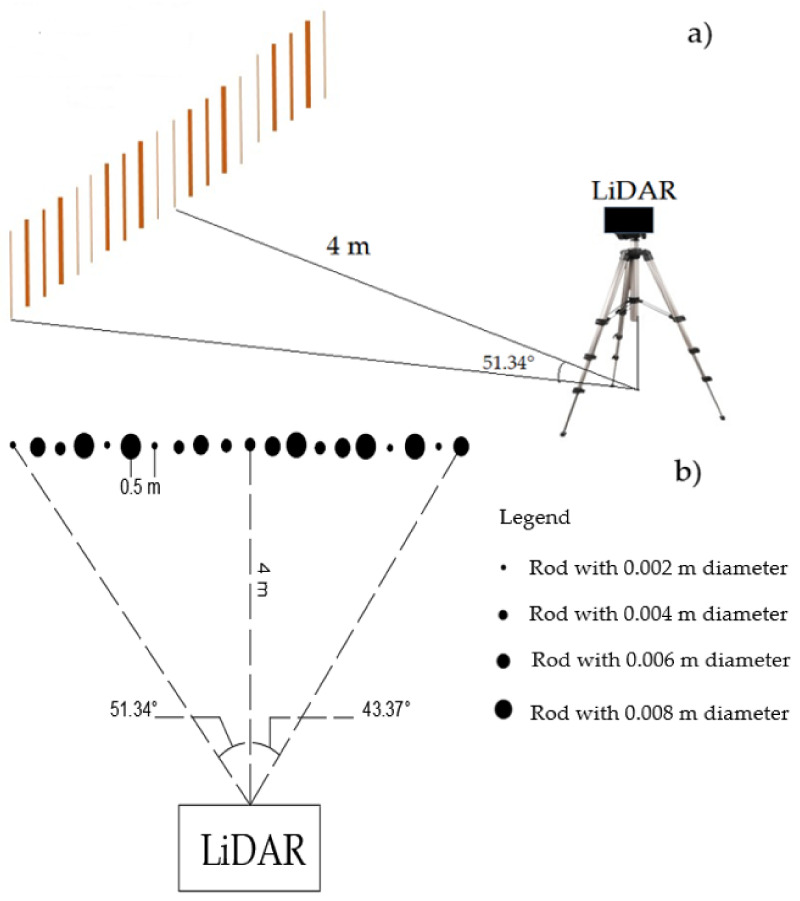
(**a**) Side-view and (**b**) bird’s-eye view of the single rods setup: 20 rods were spaced 0.5 m from each other, the LiDAR was at 4 m distance from the line of rods and pointing to the middle of the line. Black dots are symbols to show rods diameters.

In order to understand the LiDAR capability of detecting targets, individual pinewood round rods (as a proxy for weeds) at different heights (0.05, 0.10, 0.20, 0.40 or 0.60 m) were set up on a line at one of the University of Western Australia (UWA) lawns (−31.985695, 115.819597). Separate scans were done on lines of 20 wooden round rods, across the different scanning distances from the LiDAR (4, 6, 8, 10, 12, 14, 16, 18, and 20 m). The maximum distance chosen was based on approximate swath/operating width of farm machinery. The rods had different diameters (2, 4, 6 and 8 mm) that were placed at random locations across a line, spaced 0.5 m from each other (Figure 1). A total of 40 scans were performed in this trial. The same setup pattern was repeated for all 40 scans, albeit with different rod heights and distances.

The LiDAR was mounted on a stand 0.70 m above ground level, which was spread out perpendicular to the center of the LiDAR scan. The LiDAR was static and pointing to the middle rod and 10 rods were positioned on the left and nine were positioned on the right side of the LiDAR (Figure 1). The LiDAR was set at different angles below the horizontal, to view the rods. These angles were chosen based on the LiDAR distance from the line and varied between 10° and 4° below the horizontal. The angle was greater the closer the LiDAR was to the line. In these scans, the lawn represented a simplified ‘canopy’ surface and the rods represented the ‘weeds’.

#### 2.1.2. Scanning Groups of Rods

**Figure 2:**
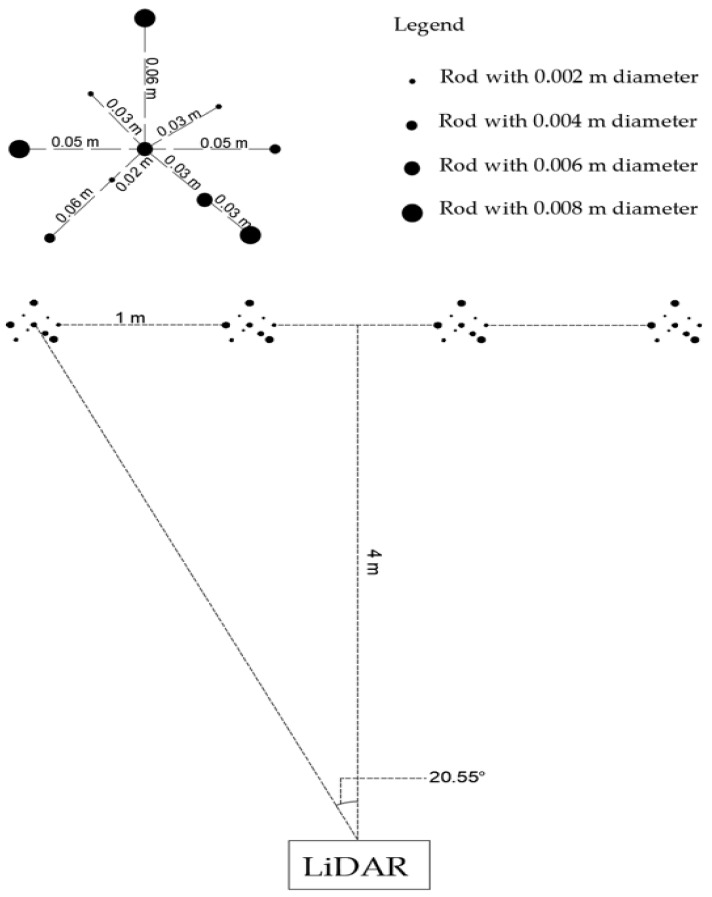
Bird’s-eye view of the group of rods trial setup. One group of rods formed a geometrical shape and rods were at the same height with different diameters. Black dots are symbols to show rod diameters. The LiDAR was placed at 4 m distance from the groups of rods and the view angle of the outermost groups of rods was 20.55 on either side of the centre ‘line’.

Since weeds tend to grow in patches [33], four groups of rods (representing weed patches) with the same shape were set up in this trial, in a line 1 m apart, at the same UWA lawn as previously described (Figure 2). Each group had 10 wooden round rods with four diameters (as described previously). Forty-five scans were performed with each having rods at one of five heights (0.05, 0.10, 0.20, 0.40 or 0.60 m) and nine distances from the LiDAR (4, 6, 8, 10, 12, 14, 16, 18 and 20 m). Individual group of rods contained three rods of each 2 mm and 8 mm diameter and two rods of each 4 mm and 6 mm diameter. Since the results of the initial ‘line of rods trial’, described previously, showed that the diameter of the rods did not affect their detection, the combination of rod diameters was chosen randomly. The LiDAR was static and mounted on a stand at 0.70 m height above the ground and was pointing between the two middle groups of rods (Figure 2). The LiDAR vertical angle was chosen as described previously and varied between 10° and 3° below the horizontal.

#### 2.1.3. Scanning Weeds in Wheat Plot

**Figure 3:**
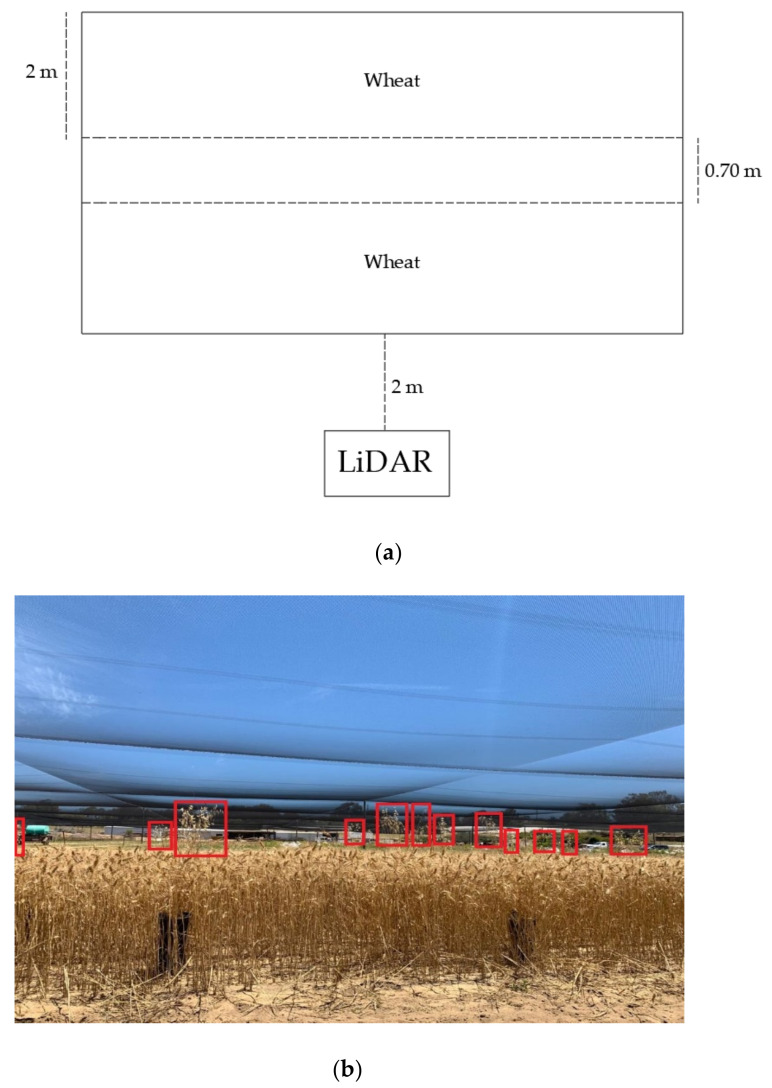
Scanning weed pots in the wheat plot, (**a**) wheat plot set up at the UWA Shenton Park Field Station (**b**) Weed pots elevated using plastic pots and randomly distributed in the wheat plot.

LiDAR scans of weeds in wheat crops were conducted at the UWA Shenton Park Field Station (−31.950012, 115.793033) in November 2019. A plot of wheat (cultivar Magenta) (45 m × 2 m) was sown in May 2019 with a row spacing of 0.20 m and a standard seed rate for Australia of 80 kg/ha (Figure 3a). Seeds of *Avena fatua* and *Sonchus oleraceus*, which were reported as the common weeds growing taller than crops in Western Australia [28], were grown in plastic pots. Seeds were placed in Petri dishes on agar and cold treated for germination. Germinated seeds were transferred to seed germination trays with potting mixture (25% peat moss, 25% sand, and 50% mulched pine bark) to be stored in a UWA glasshouse for two weeks for growth. All plants were irrigated every three days. Following four weeks of growth, one seedling was transplanted to each 0.18 m diameter plastic pot. The plants were maintained in outdoor conditions andfertilized weekly using 1 g of Scotts Cal-Mg grower plus TM soluble fertilizer [N 19% (NH2 15%, NH4 1.9%, NO3 2.1%), P 8%, K 16%, Mg 1.2%, S 3.8%, Fe 400 mg kg^−1^, Mn 200 mg kg^−1^, Zn 200 mg kg^−1^, Cu 100 mg kg^−1^, B 100 mg kg^−1^, Mo 10 mg kg^−1^] to maintain growth.

When the wheat in the plot was at Zadoks growth scale 91 [34], the pots containing *Avena fatua* and *Sonchus oleraceus* were placed into the plot on upturned empty plastic pots, ensuring that target weeds were protruding above the nominal wheat height that ranged between 0.60–0.82 m. The height of *Avena fatua* and *Sonchus oleraceus* pots ranged between 0.80–1.06 m and 0.71–0.99 m, respectively. The LiDAR was mounted on a stand at a height of 1.08 m above the ground, pointing toward the wheat at an angle of 15° below the horizontal (Figure 3b). The LiDAR was static and scanned the wheat plot at 2 m distance from the first row of wheat. Three scans were performed: the first conducted by placing 21 *Avena fatua* pots at random in the wheat as described above; the second one by using 14 *Sonchus oleraceus* pots; and the third one by placing a mixture of 14 *Avena fatua* and 9 *Sonchus oleraceus* pots in the wheat plot. At the third scan, weed pots were either placed individually or within groups (to simulate weed patches).

### 2.2. Data Acquisition and Processing

The LiDAR was connected to an ADLINK embedded computer through an Ethernet cable. The Sick Open Portal for Applications and Systems (SOPAS) Engineering Tool software was used to control the sensor and target detectability when the LiDAR was operating. The angle of the LiDAR (below horizontal) was altered before each scan, to ensure the maximum visibility of the rods in SOPAS.

The LiDAR can be controlled via the Robotic Operation System (ROS) open-source robot management software [32]. The produced ROSBAG files from the LiDAR were stored on the computer. A bag file stores ROS message data. The recorded ROSBAG files were processed in the ROS environment [35]. The GPS location of the LiDAR and the recorded point clouds during the scans were extracted from the ROSBAG files. The point clouds included the xyz coordinates and the reflected intensity values of each point. Point clouds were visualized in CloudCompare software version 2.12 alpha and the point cloud for the rods/weeds was segmented and separated by creating a moving window and each rod/weed studied individually. The moving window was aligned with the x, y, z axes and moved in all three dimensions during the segmentation. By creating a moving window, the x, y, z coordinates of rods were extracted from the point cloud. The z values were compared to the rods’ actual heights to confirm the objects were selected accurately. The actual height of the rods/weeds were compared with the estimated LiDAR data for the ‘suspected’ rods/weed to assess the accuracy of the estimated height of the targets.

Clustering was performed on the normalized reflected values from targets to understand the capability of discriminating the targets based on their return values. The reflected values in a small xy region where two different targets existed (lawn vs. rod or wheat vs. weeds) were clustered to see if two distinct clusters were detected. Clustering is one of the unsupervised data mining methods, which deals with grouping the data based on the similarity of the objects in each group cluster [36]. Silhouette measurements can be used to understand the performance of the clustering algorithms. The correctness of the assignment of the data to a particular cluster can be measured by the silhouette score. Silhouette score values vary between −1 to +1, where +1 value shows the object has been clustered correctly and −1 shows the object has not been properly clustered [37].

#### Estimating Positions of the Targets

The GPS coordinates of each target rod were recorded before each scan. The estimated GPS coordinates of each rod in trial 1 was calculated based on the GPS coordinates of the LiDAR at each scan and the estimated x and y coordinates of each rod. The accuracy of the estimated location of each rod was assessed by calculating the Euclidean distance [38]. The Euclidean distance was measured between the estimated position and the actual GPS coordinates of each rod using the Sklearn package in Python 3.7.

### 2.3. Statistical Analysis

Prior to scanning the weeds in wheat plot trial, the weeds’ heights in each scan were measured from the ground to the tallest plant part. The wheat plots’ height was determined by measuring 300 wheat plants at random across the plot. The measured wheat plants heights were tested for normality of residuals by the Shapiro-Wilk test. The mean and standard deviation (SD) of wheat crop height was calculated, with 99% of the wheat crop having a height less than the value of 3SD ± mean. This value was considered as a threshold crop height in the plot.

Binary logistic regression analysis was performed separately at each (actual) rod height, to evaluate the probability of target detection at different distances. Theis regression is a linear classifier to discriminate one class observations from the other. The distance was considered the independent variable and detection of rods was the dependent binominal variable (detected = 1, not detected = 0). The probability of detecting the target rods at different distances was determined from the logistic regression. The analysis was performed in Python 3.7 using the Sklearn Package.

The degree of association between rod/weed actual heights and the LiDAR-estimated heights was assessed by calculating the Root Mean Square Error (RMSE) of the linear regression between the actual and the LiDAR-estimated heights of targets. Normal distribution and homoscedasticity of residuals at each scan were confirmed using Shapiro-Wilk and Breusch-Pagan tests, respectively. All statistical analysis was performed in Python 3.7 using Sklearn Package.

**Figure 4:**
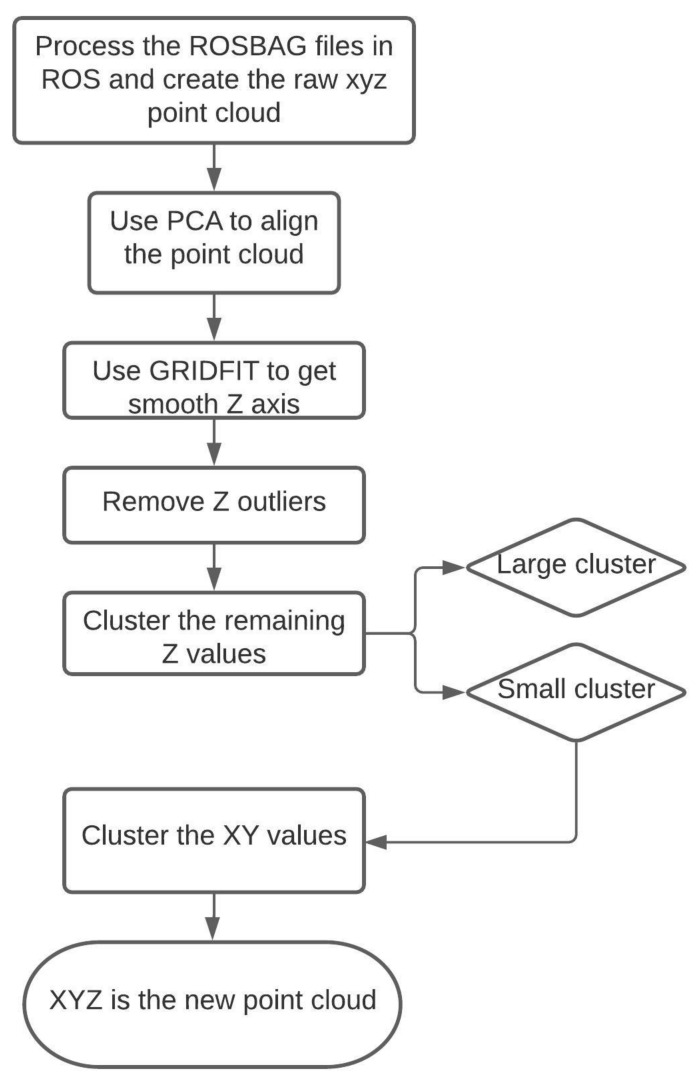
Schematic of the LiDAR data pre-processing technique.

Pre-processing steps such as removal of noise and dimensional smoothing were performed on the point cloud data (Figure 4). The dimensionality of each recorded point cloud was reduced using the principal component analysis (PCA). The analysis was performed in Python 3.7 using Sklearn package and the optimal alignment axes of the point clouds were computed within the PCA axes. After alignment, the xy axis formed the ground plane and the z-axis formed the vertical axis. The values along the aligned z-axis were smoothed using GRIDFIT surface modelling tool in MATLAB R2019b [39]. The surface modelling was created from the aligned data by fitting a surface from the form z (x, y) to the aligned data. An approximation of the raw (x, y, z) point cloud was created as the new point cloud (X, Y, Z) using surface modelling. The outliers were removed from Z values and the data was prepared for clustering.

For each trial, K-means clustering was performed on the point clouds at each scan to automate target detection and assess the accuracy of rods/weeds detection. The classical K-means clustering is based on minimizing the distance between the data points and the nearest neighbor center [40]. Furthermore, K-means clustering has the advantage of being a fast training algorithm without dependency on the order of inputting the data [41]. The (X, Y, Z) point cloud of each trial was grouped using 1-dimentional K-means clustering based on the Z values to create two major clusters of lawn and rod or wheat and weeds. The small point cloud of XYZ (rods/weeds cluster) was re-clustered (2-dimentional) to n-clusters, with n as the number of detected rods/weeds detected by moving window, based on the X and Y values. The X, Y, Z coordinates of ‘suspected’ weeds were extracted by creating a moving window from the point cloud. Cluster analysis was performed to validate and automate the weed detection process. The silhouette score was calculated to validate the n-clusters detected in each point cloud as this index was proposed by Rousseeuw [37]. Each cluster had a silhouette score, confirming the cluster separation from other clusters. The average distance between each point in one cluster was measured (a measure of cohesion) and then the average distance between that point and all points in the nearest cluster was also measured (a measure of separation from the closest other clusters). The average silhouette score was the overall average cluster coefficient calculated to confirm the clustering quality of each point cloud. Clustering was performed with n-clusters = 2, 3, …, 20 and the number of clusters with the highest silhouette score indicated a better quality of clustering results [42]. K-means clustering and silhouette analysis were performed in Python 3.7 using the Sklearn package.

## 3. Results

### 3.1. Effect of Distance on LiDAR-Estimated Height of Rods

#### 3.1.1. Scanning Lines of Rods

**Figure 5:**
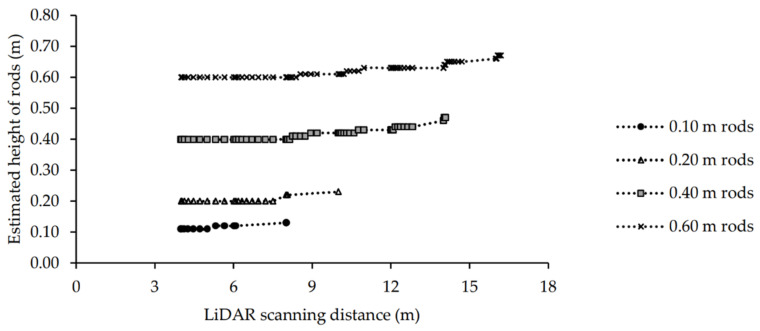
Comparison of the actual height (0.10, 0.20, 0.40 and 0.60 m) and LiDAR-estimated heights at different scanning distances of 4 to 16 m for the line of rods.

A line of individual rods was scanned by the LiDAR at different scanning distances with a range of LiDAR vertical angles. The actual heights of the rods were compared with their LiDAR-estimated heights. There was no rod detected when the LiDAR scanning distance from the line of the rods was 18 m (data not shown). The diameter of the rods did not affect their detection, and all rods with the diameter of 2 mm to 8 mm were detected by the LiDAR at < 18 m distance. The rods at 0.05 m height were not detectable at any scanning distance by the LiDAR (data not shown). As the LiDAR distance from the line of rods increased, the estimated height of the detected rods slightly increased above the actual height of the rods and the variation in the data also increased (Table 1 and Figure 5).

The results of logistic regression analysis showed that the probability of detecting a line of rods decreased with height of rod and increased distance from the LiDAR (Table 2). The probability of detecting the line of rods was < 50% for the 0.10, 0.20, 0.40 and 0.60 m high rods at 8, 10, 16 and 18 m, respectively. The RMSE for the linear regression between the actual and the LiDAR estimated heights of rods at scanning distances of 4–16 m was calculated between 0.02–0.07 m (r^2^~0.99, *p* ≤ 0.001). In addition to the LiDAR scanning distance, the position (angle) of the rods from the center ‘line’ affected detection and the estimated height of the rods. The angle to the outermost detected rods varied at each scanning distance. The angle from directly in front of the LiDAR to the outermost group of rods when the instrument was placed at the scanning distances of 4 to 16 m from the groups of rods, varied between 51.34° at 4 m to 8.88° at 16 m (Table 1). By moving the LiDAR further from the line of rods (from 4 m to 18 m at the center line), the number of visualized rods at each of the heights decreased, and the angle of detected rods decreased, becoming closer to the center ‘line’ of the LiDAR (Table 1). The effect of angle from the center line is demonstrated by having one 0.20 m rod in the 10 m (center line) scan detected, which was on the center line (i.e., 10 m) from the LiDAR, however, the furthest distance of detected rod in the 8 m (center line) scan was angled at 7.12° from the center line, which was only 8.06 m from the LiDAR (Table 1). Therefore, rods directly in front of the LiDAR were detected at longer distances. The number of detected rods at each scanning distance was also related to the target size. For instance, when the LiDAR distance from the line of rods was 4 m, all 20 rods at all heights were visible in the point cloud and the actual distance from the LiDAR to the outermost rods was 6.40 m and by increasing the LiDAR distance from the line of rods to 16 m, only 11 rods at 0.60 m were visible in the point cloud (Table 1).

In order to automate the target detection, the point cloud of each scan was clustered into the number of detected targets. The small point cloud of XYZ was re-clustered to the number of visualized rods at different height at each scan. The number of clusters was the number of detected rods in the trail. The silhouette score of this trial for clustering the point cloud to n_clusters of detected single rods by the moving window of the line of rods at different heights was between 0.38 and 0.53.

#### 3.1.2. Scanning Groups of Rods

**Figure 6:**
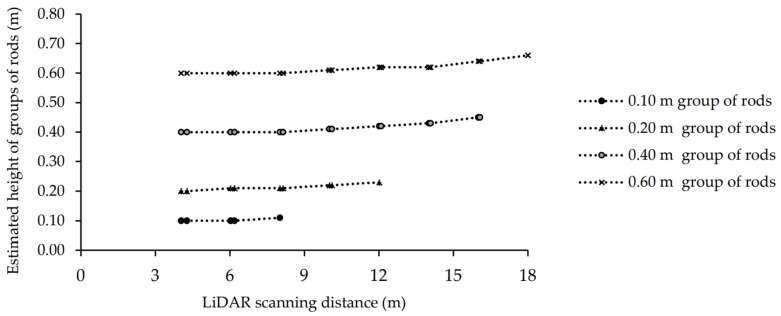
Comparison of the actual height (0.10, 0.20, 0.40 and 0.60 m) and LiDAR-estimated heights at different scanning distances of 4 to 16 m for the groups of rods.

A line of grouped rods was scanned by the LiDAR at different scanning distances with a range of LiDAR vertical angles. The actual height of each group of rods was compared to the LiDAR-estimated heights. There was no group of rods detected when the LiDAR scanning distance from the line of the rods was 20 m (data not shown). The groups of rods at 0.05 m height were not detectable at any scanning distance by the LiDAR (data not shown). As the distance of the LiDAR from the groups of rods increased, the estimated height of the rods also slightly increased above their actual height (Table 3 and Figure 6).

The RMSE for the linear regression between the actual and the LiDAR estimated heights of groups of rods at scanning distances of 4 to 18 m of rods was calculated between 0.005–0.06 m (r^2^~0.99, *p* ≤ 0.001). The angle to the outermost detected group of rods varied at each scanning distance. The angle from directly in front of the LiDAR to the outermost group of rods when the instrument was placed at the scanning distances of 4–18 m from the groups of rods was 20.55°and 1.59° on either side of the center ‘line’ (Table 3). When the LiDAR distance from the center of the line of the groups of rods was 4 and 6 m, all of the four groups of rods at all heights were visible in the point cloud, with the outermost rods at 4.27 m and 6.18 m, respectively (Table 3). (When the LiDAR distance from the groups of rods increased to 20 m there were no groups of rods detected in the point cloud (data not shown). By moving the LiDAR further from the groups of rods (from 4 m to 20 m) the number of visualized groups of rods at 0.10, 0.20, 0.40 and 0.60 m heights decreased, and the angle of detected rods decreased, becoming closer to the center ‘line’ of the LiDAR. Similar to the trial in Section 3.1.1., the point cloud of each scan was clustered into the number of detected targets to automate the target detection by the LiDAR. The small point cloud of XYZ re-clustered to 4 clusters at each scan. The number of clusters was the number of detected groups of rods in the trial. The silhouette score of this trial for clustering the point cloud to n-clusters of detected groups of rods at different height, by the moving window varied between 0.45 and 0.53 at different scanning distance. Clustering and the silhouette analysis result of the point cloud in this trial and the trial at Section 3.1.1 showed small differences existed in the thickness of the silhouette plot. In addition, all clusters had a silhouette score larger than the average score. Therefore, the similarity consistency within clusters of data by silhouette score validated the visualized number of targets in the point clouds.

### 3.2. Estimating the Location of the Targets with the LiDAR

The results showed that the location of the target with respect to LiDAR center ‘line’ was the most important factor in estimating the target location. For the 0.60 m height rods lined up 4 m from the LiDAR (distance at the center ‘line’) (Table 1), the LiDAR-estimated Euclidean distances of the rod on the center ‘line’ was 4.04 m i.e., 0.04 m further. By contrast, the outermost rod at 51.34° from the center line had an actual Euclidean distance of 6.40 m from the LiDAR, which was estimated to be 0.40 m further. This distance discrepancy for the 16 m scan was 0.08 m on the center line and compared with a discrepancy of 0.19 m for the outermost detected rod, which was 8.88° off the LiDAR center ‘line’ (Table 1). Therefore, the location discrepancy was greater further from the target and increasing angle, with the latter having the greater effect.

### 3.3. Scanning Weeds in Wheat Plot

**Figure 7:**
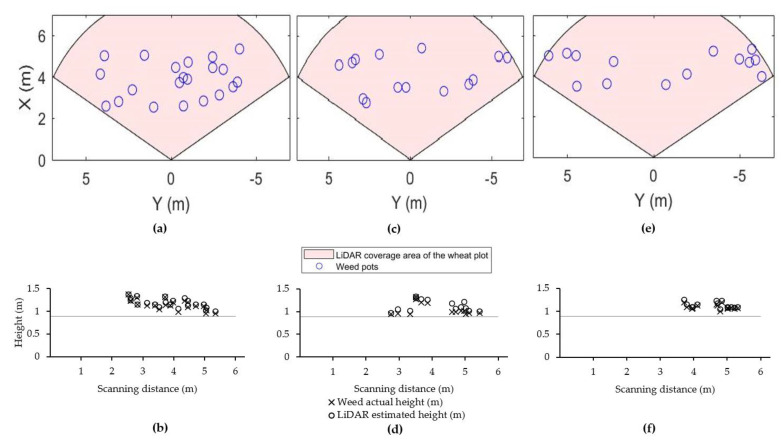
Illustration of the weed pots containing *Avena fatua* (**a**), *Sonchus oleraceus* (**c**), mix weed pots (**e**) in the wheat plot; and comparison of weed heights and the LiDAR-estimated heights for *Avena fatua* (**b**), *Sonchus oleraceus* (**d**), mix weeds (**f**). The scanning distance shows the width of the wheat plot covered by the LiDAR. The LiDAR was mounted on a stand at 1.08 m height above the ground pointing to the plot at an angle of 15° below the horizontal. The horizontal line at 0.89 m height shows the 99-percentile of wheat height.

The crop height measurements showed that 99% of the wheat plants had a height less than 0.89 m, therefore, this height was considered the top of the wheat canopy. Figure 7b,d,f) shows the comparison of the weed actual height and the LiDAR-estimated height at scanning distances between 2 m and 6 m. The RMSE between the actual and LiDAR-estimated heights of *Avena fatua*, *Sonchus oleraceus* and their combination was between 0.05 and 0.07 m (r^2^~0.99, *p* ≤ 0.001). There were 21, 14 and 23 weed pots of *Avena fatua*, *Sonchus oleraceus* and mixed weed pots, respectively, placed in the wheat plot at each scan, with 100% of the weeds in the pots detected by the LiDAR (Figure 7a,c,e).

## 4. Discussion

### 4.1. Distance and Target Size vs. Target Estimated Height

The detectability of the pinewood round rods at different scanning distances was directly influenced by the height of the target, concurring with the results of Alwan, Wagner [43]. The diameter of the target, down to 2 mm, had no effect on target detectability by the LiDAR used in this study. Targets of 0.05 m in height were not detectable from the closest distance of 4 m, however, targets 0.60 m in height were detectable from 4–16 m scanning distances. The height of the lawn (~0.03–0.05 m), into which the rods were placed, was too close to the height of the 0.05 m rods to allow separation of rods from the lawn canopy in the point cloud. The number of returning pulses per square meter from a shorter rod was less than the number of returning pulse from a taller rod to the LiDAR. Therefore, the shorter rods were detected less easily than the tall ones, at similar scanning ranges. The logistic regression results showed a lower detection rate of smaller rods at greater LiDAR scanning distances. The small RMSE between the actual and the LiDAR estimated rods heights confirmed the LiDAR as a capable device for target height estimation [44]. Furthermore, the estimated height of the targets increased above the actual height as both LiDAR horizontal scanning angle (from straight ahead) and distance of the target increased, which was also demonstrated by Alwan, Wagner [43]. Plotting the estimated height of rods at different scanning distances showed a positive height bias at all ranges, which increased non-linearly with the LiDAR range. This bias was also a function of rod height, the taller the rod and smaller the LiDAR range, the more accurate the height estimation by the LiDAR. By increasing the target distance from the LiDAR, the size of the beam footprint increases and the target should be closer to the center line of the LiDAR, to achieve the most accurate estimation of each target height [5]. The greater estimated vs. actual heights of the targets, at an increased distance from the LiDAR, could be also explained by the laser pulse divergence with distance [45]. When the LiDAR distance from the target increased, the laser pulse likely stretched in the point cloud and the size of the point became larger due to increasing the incident angle [45,46]. Moving the LiDAR further from the targets resulted in a decrease in the number of visualized groups and the angle of the detected targets decreased, becoming closer to the center of the LiDAR scan. This study showed that the height data recorded by the LiDAR could be used to detect targets and estimate their heights with accuracy ranging from ±0.019 m at 4 m distance to ±0.068 m at 16 m distance [47].

Scanning weed targets within the wheat plot near harvest time showed that 100% of the weed targets, which were projecting above the wheat crop, were detected by the LiDAR during the scans with RMSE of 0.05–0.07 m between the actual and estimated height of weeds.

Similar results were found by Andújar et al. [5] for detecting early-season weed species using a terrestrial LiDAR sensor pointing to the inter-row area. In the current study, the wheat plots were relatively mall, and the weeds were maximum of ~6 m from the LiDAR, however, the earlier studies with rods showed that targets projecting 0.60 m above the crop could be detected up to 16 m from the LiDAR.

The basic principles of the capability of the 3D scanning LiDAR for target detection (i.e., projection above the crop canopy) in this study were: (1) target size played a significant role in the detectability of the target. The smaller the size of the target and the further the LiDAR scanning distance, the probability of detecting the target was reduced, (2) individual targets with height ≤ 0.60 m were not detectable at distances ≥ 18 m and grouped targets, simulating weed patches, with height ≤ 0.60 were not detectable at distance ≥ 20 m, (3) targets with height ≤ 0 05 m were not detectable with the sensor, (4) targets with diameter ≥ 2 mm were detectable, (5) the location estimation by the LiDAR was more accurate when the target was closer to LiDAR center ‘line’ compared with away from the center ‘line’. Overall, detection of target/weeds by the LiDAR was a function of the LiDAR scanning range, weed size, LiDAR noise characteristics and its detection scheme.

### 4.2. Estimating of the Target Location

Similar to the target height estimation by the LiDAR, results showed that when the target was detected by the LiDAR the critical factor for accurately estimating the location of the target by the LiDAR was the target horizontal angle from the LiDAR center ‘line’. This can be explained by the reflection intensity data that LiDAR receives from the target. The strength of the returned laser beam from the target depends on the LiDAR scanning distance from the target and the angle between the target surface and the LiDAR beam [48]. Franceschi et al. [49] and Jelalian [50] reported the 1/R^2^ reflected intensity vs. distance relationship, at longer distances beams are returned at a lower strength and the reflected intensity is reduced. Therefore, the accuracy of the estimated location of the target is a combination of different factors such as the target size, LiDAR scanning distance [20], and target orientation toward the LiDAR center ‘line’.

Detecting weeds using sensors such as LiDAR is an evolutionary step in SSWM. Nowadays, early-season weed detection in conjunction with real-time weed control is becoming widespread in broadacre cropping fields with herbicide reduction of up to 90% [51]. Attaching the sensors to farming machinery at harvest in conjunction with a GPS unit for mapping can lead to an efficient basis for SSWM in the following season as weed seeds entering the soil weed seed bank will pose a threat to successive field crops on that location [52]. SSWM based on sensor technologies to create agronomically informed prescription maps have resulted in a significant reduction in herbicide use [53]. Since weed distribution of many species is typically sporadic or patchy, the successful mapping of weed patches, using the data collected from ground-based sensors and analyzed by machine learning techniques, can provide important information that allows the more judicious use of weed management inputs [54].

The LiDAR detection performance can be affected by certain weather conditions such as heavy snow/rain or dense fog [55] and some methods such as classification of weather condition [56] or de-noising the LiDAR point cloud [57] can improve LiDAR performance. The SICK MRS6000 LiDAR used in this study has a mirror technology that can simultaneously multiply the 3D point cloud density without any gaps in rainy and foggy conditions [32].

The repeated use of herbicide input has led to the widespread proliferation of herbicide-resistant weeds within a crop field [58]. Therefore, SSWM methods such as sensors to detect and monitor weed patches have the potential to save a considerable amount of herbicide inputs and are becoming a promising weed control method [59]. Furthermore, since the herbicide-resistant weed patches can proliferate across fields, diverse integrated weed management practices are necessary for maintaining herbicide effectiveness [58]. Therefore, SSWM methods such as sensors to detect and monitor weed patches have the potential to save a considerable amount of herbicide inputs and are becoming a promising weed control method [29]. The success of these methods depends on the efficiency of the sensor, the accuracy of the collected data by the sensor and its strength in detecting weeds [60]. The use of weed detecting sensors such as LiDAR provides an important tool in detecting weed patches before they proliferate across the entire field. This study demonstrates that LiDAR sensors can be considered as a potential valuable technique for automated weed detection and location, based on their height difference from the crop canopy. Persistent weed species such as *Avena fatua* and *Sonchus oleraceus* which tends to persist in the field until harvest, grow taller than the crop and shed their seeds before harvest [28] could be the potential targets of this method.

## 5. Conclusions

In this work, a 3D LiDAR scanner was used to assess the LiDAR capability of detecting targets and to understand the potential of this technology for detecting and estimating the position of weeds that grow taller than the crop canopy near crop maturity. The results of the study demonstrate the potential of LiDAR sensors for detecting and estimating the height and position of weed species within cropping fields. This information can be used to map the location of weeds that escape early weed control and persist until harvest, to develop a long-term weed management strategy for the coming seasons. Furthermore, farmers can evaluate the efficiency of their previous weed control methods by mapping and monitoring changes in the weed patches. New and improved, economical, weed detection systems with high accuracy and efficiency are continually required to increase the quality and quantity of the information for growers to manage economically damaging weed populations. The increasing prevalence of multiple herbicide-resistant weed species, means an increased effort is needed to develop new and improved weed detection and mapping technologies, as a basis to automate the application of diverse integrated weed management systems. The results of this study suggest that ground-based LiDAR sensors have the potential to detect and map populations of weed species that are continually evolving or adapting in order to survive within agricultural systems.

## Figures and Tables

**Table 1:** Effect of LiDAR distance on detectability and height estimation of a perpendicular line of 20 rods. The LiDAR was 0.70 m above the ground. The LiDAR distance from the middle of the line of rods was 4, 6, 8, 10, 12, 14, 16 m and the LiDAR vertical angle was 10°, 9°, 7°, 7°, 7°, 5°, 4°, respectively.

	Rods Actual Height (m)	Number of Detected Rods	Mean Estimated Height (m)	SD of Estimated Heights (m)	LiDAR Angle to the Outermost Detected Rods (Right-Left)	LiDAR Distance from the Outermost Detected Rod (m)
4 m (10°)	0.10	20	0.12	±0.014	51.34° to −43.37°	6.40
	0.20	20	0.20	±0.015	51.34° to −43.37°	6.40
	0.40	20	0.40	±0.014	51.34° to −43.37°	6.40
	0.60	20	0.60	±0.024	51.34° to −43.37°	6.40
6 m (9°)	0.10	5	0.11	±0.006	9.45° to −9.45°	6.08
	0.20	20	0.20	±0.006	39.80° to −36.87°	7.80
	0.40	20	0.40	±0.008	39.80° to −36.87°	7.80
	0.60	20	0.60	±0.012	39.80° to −36.87°	7.80
8 m (7°)	0.10	3	0.13	±0.009	3.58° to −3.58°	8.01
	0.20	5	0.22	±0.012	7.12° to −7.12°	8.06
	0.40	20	0.41	±0.008	32.00° to −29.36°	9.43
	0.60	20	0.61	±0.010	32.00° to −29.36°	9.43
10 m (7°)	0.20	1	0.23	N/A	0	10.00
	0.40	20	0.42	±0.019	26.56° to −24.23°	11.18
	0.60	20	0.62	±0.020	26.56° to −24.23°	11.18
12 m (7°)	0.40	20	0.44	±0.019	22.64° to −20.55°	13.00
	0.60	20	0.63	±0.015	22.64° to −20.55°	13.00
14 m (5°)	0.40	7	0.47	±0.019	6.11° to −6.11°	14.08
	0.60	20	0.65	±0.021	19.80° to −17.74°	14.86
16 m (4°)	0.60	11	0.67	±0.015	8.88° to −8.88°	16.19

**Table 2:** Probability of the LiDAR detecting target rods of different heights at scanning distances of 4–18 m. A total of 20 rods at each height were scanned by the LiDAR at each distance.

Rod’s Height (m)	Scanning Distances of the LiDAR (m)							
	4	6	8	10	12	14	16	18
0.10	0.99	0.74	0.10	0.07	0.00	0.00	0.00	0.00
0.20	0.99	0.98	0.57	0.20	0.00	0.00	0.00	0.00
0.40	1.00	1.00	0.99	0.99	0.99	0.51	0.01	0.00
0.60	1.00	1.00	1.00	1.00	0.99	0.99	0.68	0.2

**Table 3:** Effect of LiDAR distance on detectability and height estimation of a perpendicular line of four groups of rods. The LiDAR was 0.70 m above the ground. The LiDAR distance from the middle of the line of rods groups was 4, 6, 8, 10, 12, 14, 16, 18 m and the LiDAR vertical angle was 10°, 9°, 7°, 7°, 7°, 5°, 4°, 4°, respectively.

	Group of Rods Actual Height (m)	Number of Detected Group of Rods	Mean Estimated Heights (m)	SD of Estimated Heights (m)	LiDAR Angle to the Outermost Detected Group of Rods (Right-Left)	LiDAR Distance from the Outermost Detected Group of Rods (m)
4 m (10°)	0.10	4	0.10	±0.002	20.55° to −20.55°	4.27
	0.20	4	0.20	±0.006	20.55° to −20.55°	4.27
	0.40	4	0.40	±0.006	20.55° to −20.55°	4.27
	0.60	4	0.60	±0.003	20.55° to −20.55°	4.27
6 m (9°)	0.10	4	0.10	±0.003	14.04° to −14.04°	6.18
	0.20	4	0.21	±0.005	14.04° to −14.04°	6.18
	0.40	4	0.40	±0.002	14.04° to −14.04°	6.18
	0.60	4	0.60	±0.0008	14.04° to −14.04°	6.18
8 m (7°)	0.10	2	0.11	±0.001	3.57° to −3.57°	8.02
	0.20	4	0.21	±0.009	10.62° to −10.62°	8.14
	0.40	4	0.40	±0.006	10.62°to −10.62°	8.14
	0.60	4	0.60	±0.005	10.62°to −10.62°	8.14
10 m (7°)	0.20	4	0.22	±0.011	8.53° to −8.53°	10.11
	0.40	4	0.41	±0.006	8.53° to −8.53°	10.11
	0.60	4	0.61	±0.007	8.53° to −8.53°	10.11
12 m (7°)	0.20	4	0.23	±0.009	7.12° to −7.12°	12.09
	0.40	4	0.42	±0.010	7.12° to −7.12°	12.09
	0.60	4	0.62	±0.010	7.12° to −7.12°	12.09
14 m (5°)	0.40	4	0.43	±0.013	6.11° to −6.11°	14.08
	0.60	4	0.62	±0.008	6.11° to −6.11°	14.08
16 m (4°)	0.40	4	0.46	±0.025	5.35° to −5.35°	16.07
	0.60	4	0.64	±0.005	5.35° to −5.35°	16.07
18 m (4°)	0.60	2	0.66	N/A	1.59° to −1.59°	18.06

## Data Availability

Restrictions apply to the availability of these data. The data are not publicly available due to third party involvement and ethical reasons.
